# Evaluating Changes in Omega-3 Fatty Acid Intake after Receiving Personal *FADS1* Genetic Information: A Randomized Nutrigenetic Intervention

**DOI:** 10.3390/nu9030240

**Published:** 2017-03-06

**Authors:** Kaitlin Roke, Kathryn Walton, Shannon L. Klingel, Amber Harnett, Sanjeena Subedi, Jess Haines, David M. Mutch

**Affiliations:** 1 Department of Human Health and Nutritional Sciences, University of Guelph, Guelph, ON N1G 2W1, Canada; kroke@uoguelph.ca (K.R.); sklingel@uoguelph.ca (S.L.K.); aharnett@uoguelph.ca (A.H.); 2 Department of Family Relations and Applied Nutrition, University of Guelph, Guelph, ON N1G 2W1, Canada; kwalton@uoguelph.ca (K.W.); jhaines@uoguelph.ca (J.H.); 3 Department of Mathematics and Statistics, University of Guelph, Guelph, ON N1G 2W1, Canada; sdang@binghamton.edu

**Keywords:** omega-3 fats, nutrigenomics, personalized nutrition, eicosapentaenoic acid, EPA, docosahexaenoic acid, DHA, single nucleotide polymorphisms, SNPs, fatty acid desaturase 1

## Abstract

Nutrigenetics research is anticipated to lay the foundation for personalized dietary recommendations; however, it remains unclear if providing individuals with their personal genetic information changes dietary behaviors. Our objective was to evaluate if providing information for a common variant in the fatty acid desaturase 1 (*FADS1*) gene changed omega-3 fatty acid (FA) intake and blood levels in young female adults (18–25 years). Participants were randomized into Genetic (intervention) and Non-Genetic (control) groups, with measurements taken at Baseline and Final (12 weeks). Dietary intake of eicosapentaenoic acid (EPA) and docosahexaenoic acid (DHA) was assessed using an omega-3 food frequency questionnaire. Red blood cell (RBC) FA content was quantified by gas chromatography. Implications of participation in a nutrigenetics study and awareness of omega-3 FAs were assessed with online questionnaires. Upon completion of the study, EPA and DHA intake increased significantly (*p* = 1.0 × 10^−4^) in all participants. This change was reflected by small increases in RBC %EPA. Participants in the Genetic group showed increased awareness of omega-3 terminology by the end of the study, reported that the dietary recommendations were more useful, and rated cost as a barrier to omega-3 consumption less often than those in the Non-Genetic group. Providing participants *FADS1* genetic information did not appear to influence omega-3 intake during the 12 weeks, but did change perceptions and behaviors related to omega-3 FAs in this timeframe.

## 1. Introduction

The field of nutritional genomics, or nutrigenetics, aims to unravel the genetic basis for why individuals respond differently to the same nutrients and/or foods [1,2,3]. The long-term outcomes of this research are expected to lay the foundation for personalized dietary recommendations to help prevent the development of chronic diseases. A more direct outcome of nutrigenetics research may simply entail the use of personal genetic information as an additional factor to help motivate people to adopt healthier dietary behaviors.

To date, the vast majority of nutrigenetic studies have examined and assessed perceptions of genetic and health information in various populations [3,4,5]. When individuals were queried, many reported to be interested in undergoing genetic testing for the prevention of chronic diseases [6,7,8,9]. However, there are currently a limited number of randomized controlled nutrigenetic trials assessing if providing genetic information actually changes dietary behaviors. The few nutrigenetic intervention studies performed to date suggest that individuals who receive personal genetic information may make more changes to their diet compared to controls [10,11,12,13].

Omega-3 fatty acids (FAs) represent an ideal nutrient to examine in the context of a nutrigenetics intervention for several reasons. From a global health perspective, it is widely recognized that increased intake of omega-3 FAs, in particular eicosapentaenoic acid (EPA, 20:5*n*-3) and docosahexaenoic acid (DHA, 22:6*n*-3), is beneficial for cardiovascular, metabolic, developmental and cognitive health [14,15,16]. However, the consumption of EPA- and DHA-rich foods such as fatty fish is low, specifically within the Western diet [17,18]. Therefore, finding new ways to motivate people to increase their consumption of omega-3 FAs are necessary.

From a genetic perspective, EPA and DHA are endogenously produced to a limited extent through a well-characterized pathway that desaturates and elongates the essential omega-3 FA, alpha-linolenic acid (ALA, 18:3*n*-3). The fatty acid desaturase 1 and 2 genes (*FADS1* and *FADS2*) play critical roles in this pathway [19,20]. It has consistently been demonstrated that single nucleotide polymorphisms (SNPs) in the *FADS* genes influence the degree of endogenous conversion of ALA into EPA and DHA [21,22]. Specifically, individuals who carry the minor allele in one or more SNP(s) in *FADS1* and/or *FADS*2 have been reported to have reduced desaturase activity, resulting in lower levels of EPA [21,22]. Gillingham et al. showed that when minor allele carriers were provided dietary ALA, blood EPA levels were increased to a level equivalent to that observed in major allele carriers [23]. This suggests that giving individuals their personal *FADS* genotype information may yield a new approach to encourage increased intake and optimize dietary recommendations for omega-3 FAs.

Providing personal genetic information in relation to omega-3 FAs in the context of a randomized intervention represents a novel area of investigation. Therefore, the objective of this study was to test the impact of providing personal genetic information for *FADS1* on the consumption of omega-3 FAs in a population of female adults over a 12-week period. We examined changes in EPA and DHA intake from foods and supplements, analyzed blood omega-3 FA levels, and assessed perceptions of nutrition and genetics.

## 2. Materials and Methods

### 2.1. Participants and Ethics

**Figure 1:**
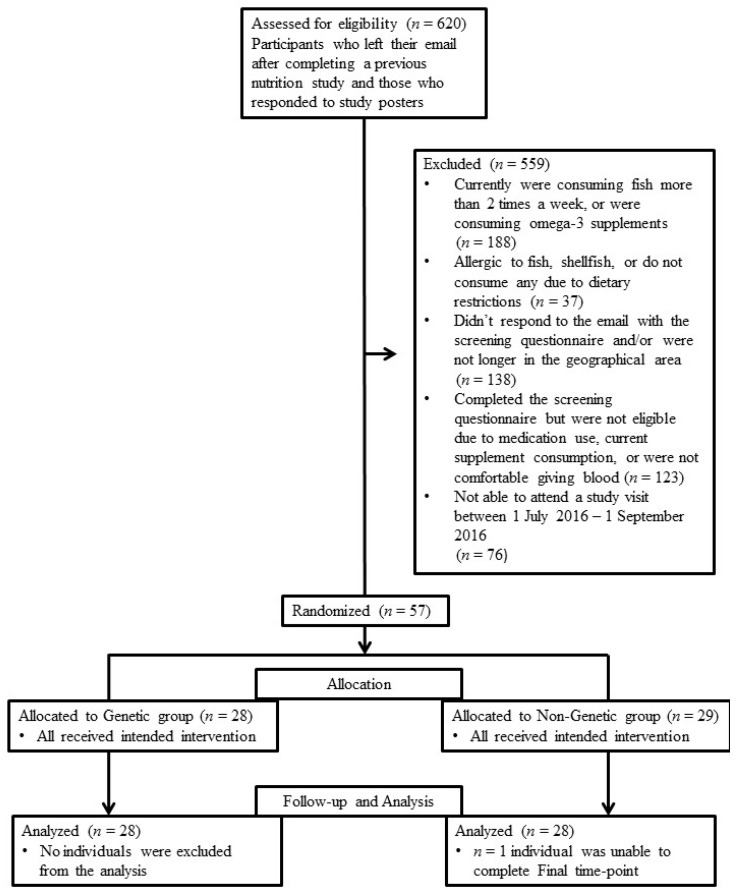
Study flow chart. Consolidated Standards of Reporting Trials (CONSORT) guidelines were used for reporting.

Female adults (between 18–25 years) were recruited through emails and poster advertisements displayed around the University of Guelph campus. Individuals completed an online screening questionnaire prior to acceptance into the study. Individuals were ineligible to participate in the study if they regularly consumed omega-3 FA supplements and/or fish more than two times per week. Baseline and Final (week 12) study visits were scheduled when participants were menstruating to minimize variability in lipid levels within an individual, as recommended by Mumford et al. [24]. Therefore, participants who reported having a regular menstrual cycle in the online screening questionnaire and would expect their period (approximately) every 28 days were eligible for the study. Study design and participant flow through the study is reported using Consolidated Standards of Reporting Trials (CONSORT) guidelines (Figure 1). Ethical approval for the study was granted by the University of Guelph Human Research Ethics Board (REB#:15AP019). The trial was registered at clinicaltrials.gov (NCT02829138).

### 2.2. Study Design

Participants attended three in-person study visits. Participants signed the consent form and provided a saliva sample to be used for DNA analysis at visit #1. Once all DNA samples were analyzed, participants were randomized into either a Genetic group (intervention) or Non-Genetic group (control) using a random number generator by two individuals external to the study intervention. The randomization process used also ensured that each group would have an equivalent number of major and minor allele carriers. The lead investigator for the study intervention was blinded to this assignment. Participants were informed of which group they were in at Visit #2.

Visit #2 corresponded to the baseline study visit. Participants provided fasted blood samples, had a one-on-one information session, and received a copy of all study intervention materials. At this visit, all participants were also given a generic information document that provided details of omega-3 FAs, and an overview of the association between SNPs in *FADS1* and omega-3 metabolism. Specifically, this document provided information about omega-3 FAs, including general nutritional information, foods and supplements that have these FAs, and possible health effects associated with their consumption; compiled from the Dietitians of Canada fact sheets [25,26]. Furthermore, the document also provided a brief overview of the reported difference in omega-3 FA levels in relation to a common SNP in *FADS1* (*rs174537*). Specifically, it was indicated that individuals who are homozygote GG allele carriers have been reported to have more EPA in their bodies and an increased ability to convert ALA into EPA and DHA [27,28,29], while individuals with at least one copy of the minor allele (GT or TT) were shown to have less EPA in their bodies and a reduced ability to convert ALA into EPA and DHA [21,22]. This generic information document was provided to all study participants to ensure that individuals in both the Genetic and Non-Genetic groups had a similar level of knowledge regarding omega-3 FAs and the influence of genetic variation in *FADS1*.

At the end of Visit #2, each participant was given a security-style sealed envelope to open after their appointment that made them aware if they had been randomly selected to the Genetic Group or the Non-Genetic Group. If participants were assigned to the Genetic group, the letter identified them as either a major (GG) or minor (GT + TT) allele carrier, according to their personal *FADS1* genotype. If the participants were in the Non-Genetic group, the letter indicated that they would receive their genetic information at the end of the study. Importantly, we did not provide any dietary advice in a genotype-specific manner, as the primary goal of the study was to assess if individuals who were given their personal *FADS1* genetic information changed their omega-3 dietary habits or not.

Visit #3 corresponded to the final week 12 study visit. All participants provided a fasted blood sample. Participants in the Non-Genetic group were given their personal *FADS1* genetic information at this point.

Participants were contacted by email throughout the study for survey distribution and appointment scheduling. Participants were not provided with additional omega-3 FA or genetic information throughout the intervention; however, we acknowledge it is possible that participants may have sought out more information independently (not monitored by the research team).

### 2.3. Online Surveys

Participants completed online food frequency questionnaires (FFQs) at Baseline and Final (week 12) to assess dietary intake of omega-3 FAs. Data on the awareness of omega-3 FAs, perceptions of receiving genetic information, and overall perceptions of the study intervention was also assessed through online questionnaires at Final (week 12). Qualtrics software (V13.28.05, ©2015, Provo, UT, USA) was used to host the online surveys. The online surveys were pilot-tested to ensure ease of completion. Participants could complete the surveys within a week of their distribution, on an electronic device of their choosing. There were questions of various types (multiple choice, select all that apply, Likert-style rating questions, sliding bar scales, and open text boxes). Survey questions are provided in Supplementary File.

#### 2.3.1. Food Frequency Questionnaires

Dietary intake of omega-3 FAs (specifically EPA and DHA) was assessed at Baseline and Final (week 12) using a validated Canadian FFQ [30] that was updated to be reflective of the omega-3 enriched foods currently available on the market (e.g., newer brands of eggs and spreads fortified with EPA and DHA). This FFQ was then translated to an online version for participant completion. For each category of whole food, functional food, or supplement, the FFQ prompted specific product names/brands, frequency of consumption, and portion size consumed during the past week. The Canadian Nutrient File (version 2015) was used to assess the amount of EPA and DHA in whole foods (e.g., fish, eggs, poultry) [31]. Researchers used food labels obtained from Internet searches and conducted visits to local grocery stores to confirm amounts of omega-3 FAs in functional foods and supplements enriched with EPA and DHA. Many food products do not distinguish between the amount of EPA or DHA within a food product, and thus EPA and DHA are represented together as a summation of both FAs. To reflect the current Canadian dietary recommendations for both EPA and DHA, the total daily intake of EPA and DHA was combined and calculated for each participant in this analysis.

#### 2.3.2. Diet and Genetics Questionnaire

This survey was comprised of researcher-generated and literature-generated questions. We examined the participants’ awareness of terminology used to describe omega-3 FAs, including the full scientific names (alpha-linolenic acid, eicosapentaenoic acid, docosahexaenoic acid) and their corresponding abbreviations (ALA, EPA, and DHA), at the start and end of the intervention. After the final study visit, participants completed a questionnaire regarding perceived dietary changes and perceptions of the study intervention (questions in Supplementary File). Questions that focused on general perceptions in nutrition and health were incorporated based on the work from the Canadian Behaviour, Attitude and Nutrition Knowledge Survey (BANKS) [32]. Additionally, participants in the Genetic group received questions regarding perceptions of genetic information, which included some questions from a nutrigenomics survey previously developed by Nielsen and El-Sohemy [33].

### 2.4. Experimental Procedures

#### 2.4.1. Genotyping

DNA was extracted from saliva using the Oragene DNA collection kit, according to manufacturer’s instructions (DNA Genotek, Ottawa, ON, Canada). Participants were genotyped for the rs174537 SNP in *FADS1* using a validated TaqMan genotyping assay (Life Technologies, Carlsbad, CA, USA). This SNP was first reported in a large genome wide association study [21], and has been consistently reported by both our lab [34,35] and others [22,27,36,37,38] to influence blood FAs and estimated desaturase activity. We used a dominant model to group individuals carrying at least one copy of the minor allele (GT or TT) into a single group (GT + TT). The association between *rs174537* and estimated desaturase activity was confirmed in the present study [39].

#### 2.4.2. Gas Chromatography for FA Analysis

Following an overnight fast, venous blood samples were collected from participants at Baseline and Final (week 12) visits. Serum and red blood cells (RBCs) were separated by centrifugation and frozen at −80 °C for subsequent analyses. RBC FAs were extracted with chloroform:methanol (2:1, *v*/*v*), using the methodology established by Folch et al. [40]. Gas chromatography was performed as previously described [41], with only minor modifications. Briefly, frozen RBC samples were thawed on ice for approximately 1.5 h prior to extraction. After the addition of 10 μL (0.1 mg/mL stock) of an internal free FA standard (C17:0), total lipids were extracted from 100 μL of RBC. The next day, samples were centrifuged at 1460 rpm for 10 min. The extraction process was repeated once using an equivalent volume of chloroform. Pooled lipids were saponified using 2 mL of 0.5 mol/L KOH in methanol at 100 °C for 1 h. The resulting free FAs were trans-esterified at 100 °C for 1.5 h. The organic phase was extracted, evaporated under nitrogen gas, and reconstituted in 100 μL of hexane for analysis. FA methyl esters were separated by gas chromatography using an Agilent 6890B gas chromatograph (Agilent Technologies, Santa Clara, CA, USA). FAs of interest were reported as percent FA composition: ALA, EPA, DHA, linoleic acid (LA; 18:2*n*-6) and arachidonic acid (AA; 20:4*n*-6). The Omega-3 Index was calculated by summing %EPA and %DHA [42]. FADS pathway activity was estimated by dividing %AA/%LA, as previously reported [27,36,41].

#### 2.4.3. Clinical Measurements

Following an overnight fast, venous blood samples were collected from participants at Baseline and Final (week 12) visits. Fasted serum samples were sent to LifeLabs Medical Laboratory Services (Guelph, ON, Canada) for the analysis of triglycerides (TAG), total cholesterol, LDL-cholesterol (LDL-c), and HDL-cholesterol (HDL-c).

### 2.5. Statistics

*R* software (R Core Team, Version 3.3.0, Vienna, Austria) was used to determine a sufficient sample size prior to commencing the study. A minimum sample size of *n* = 25 individuals per group was calculated using changes in RBC %EPA reported in previous omega-3 FA intervention studies, and differences in RBC %EPA based on SNPs in *FADS1*. The %EPA values used to calculate the effect sizes were based on past work from our lab [34,43] and others [28,44]. Sample size was determined using an alpha level of 0.05 and a power level of 0.8. Intention to treat analysis was implemented (CONSORT), where participants remained in their original intervention groups throughout data analysis.

All survey data including FFQs, and diet and genetics questionnaires were analyzed using SPSS (IBM Corporation, Version 23, Armonk, North Castle, NY, USA). Descriptive statistics were completed for all questions to determine trends and potential issues before individual question analysis. A repeated measures two-way analysis of variance (ANOVA) assuming unequal variance was used to analyze dietary intakes of EPA and DHA using Group (Genetic vs. Non-Genetic), Time (Baseline vs. Final), and the Group × Time interaction. As a secondary outcome, major (GG) and minor (GT + TT) allele carriers within the Genetic group were also compared to determine any differences related to genotype. A repeated measures two-way ANOVA was also used to analyze awareness of omega-3 terminology, using Group and Time effects as described above. Pearson’s χ^2^ tests were used for questions requiring a determination of difference in proportions between the Genetic and Non-Genetic groups. An independent samples *t*-test was used to determine differences in the means between the Genetic and Non-Genetic groups when analyzing Likert-style questions (scale from 1–7).

GraphPad Prism (GraphPad Software, Version 6, La Jolla, CA, USA) was used to assess normality of the data, and to evaluate differences in anthropometric, clinical, and FA data at Baseline and Final with a repeated measures two-way ANOVA, using Group, Time and Group × Time effects, as described above. A *p* ≤ 0.05 was considered statistically significant. *R* software (R Core Team, Version 3.3.0, Vienna, Austria) was used for correction of multiple comparisons using the Benjamini Hochberg approach [45,46].

## 3. Results

### 3.1. Participant Characteristics

The age of participants ranged from 19–25 years (mean = 22.0 ± 1.5 years). On average, participants rated interest in their personal health as 8.5 ± 1.3, out of a possible score of 10. Self-selected ethnicity, school or employment status, field of study/area of work, and contraceptive use were not different between the Genetic and Non-Genetic groups (Table 1).

All participants were genotyped for *rs174537* in *FADS1*. Our population consisted of 26 homozygous major (GG), 23 heterozygous (GT), and 8 homozygous minor allele (TT) carriers (Table 1). The SNP was confirmed to be in Hardy-Weinberg equilibrium. Hereon, we refer to individuals with the GG genotype as “major allele carriers” and individuals with either GT or TT genotypes as “minor allele carriers”. Genotypes were evenly and randomly divided between the intervention and control groups.

### 3.2. FFQ Analysis

At baseline, participants were consuming an average of 200 ± 29 mg EPA and DHA/day. There were no significant differences in EPA and DHA intake between Genetic and Non-Genetic groups at Baseline (Table 2). As indicated by the Group × Time interaction, the nutrigenetic intervention did not differentially influence dietary intake of EPA and DHA (*p* = 0.27) (Table 2); however, both groups significantly increased their intake of EPA and DHA during the intervention (*p* = 1.0 × 10^−4^).

The Genetic group was further investigated to determine if there were any differences in EPA and DHA consumption when stratified according to *FADS1* genotype. Dietary intake of EPA and DHA tended to be higher in GT + TT individuals (272 ± 71 mg/day) than GG individuals (141 ± 38 mg/day) at Baseline (*p* = 0.06). The same trend was seen at final (week 12), where dietary intake of EPA and DHA was higher in GT + TT individuals (432 ± 112 mg/day; ~59% higher than Baseline) than GG individuals (198 ± 34 mg/day; ~40% higher than Baseline) (*p* = 0.08). However, the Group × Time interaction revealed there was no significant difference in EPA and DHA intake between GG and GT + TT individuals at Final (week 12) (*p* = 0.29). 

There were 10/56 (~18%) participants (four in the Genetic Group and six in the Non-Genetic Group) who reported initiating supplement use after the first study visit and who continued taking these supplements up to week 12. Out of these 10 participants, nine participants took fish oil supplements and one participant took an algae oil supplement. These participants were amongst those with the highest EPA and DHA intakes measured with the FFQ at Final (week 12).

### 3.3. FA Analysis 

There were no differences between the Genetic and Non-Genetic groups at Baseline for %ALA, %DHA or the Omega-3 Index (Table 2). %EPA was higher in the Non-Genetic group compared to the Genetic group at Baseline. At the end of the study intervention, %EPA increased similarly in both groups (*p* = 0.02) (Table 2). The Omega-3 Index also showed a significant Time effect. The Group × Time interaction analysis revealed the nutrigenetic intervention had no differential effect on these FAs (Table 2).

### 3.4. Clinical Blood Lipid Analysis

There were no differences between the Genetic and Non-Genetic groups at Baseline for any of the clinical parameters measured (Table 2). As indicated by the Group × Time interaction, the nutrigenetic intervention did not differentially affect any of the parameters between the two groups (Table 2). However, both groups experienced small increases in Total cholesterol, the Chol/HDL ratio, LDL and Non-HDL from Baseline to Final (Table 2). The change in the Chol/HDL ratio was not significant after correction for multiple comparisons.

### 3.5. Diet and Genetics Questionnaires

#### 3.5.1. Awareness of Omega-3 FA Terminology

The Genetic and Non-Genetic groups were well matched for their level of awareness of omega-3 terminology at Baseline (Table 2, Supplementary File: Q1). A group effect was observed regarding the awareness of docosahexaenoic acid, where more individuals in the Genetic group said they were familiar with the term at Final (week 12) compared to the Non-Genetic group (Table 2). Awareness of both abbreviations and full scientific names increased in both groups during the intervention and remained significant after correction for multiple testing (Table 2). Lastly, Group × Time interactions were observed for both the ALA and EPA abbreviations, with individuals in the Genetic group reporting greater awareness of these terms compared to the Non-Genetic group.

#### 3.5.2. Perceptions and Use of Generic Omega-3 Nutritional Information

Overall, there was a significant difference in perceptions of the study intervention between the Genetic and Non-Genetic groups at Final (week 12) (Table 3, Supplementary File: Q2). Specifically, the Genetic group reported to agree more strongly that the generic omega-3 FA information document was new to them (*p* = 0.05) and that this information was useful when they considered their diet throughout the study (*p* = 0.03), in comparison to the Non-Genetic group (Table 3).

#### 3.5.3. Perceived Dietary Changes

At Final (week 12), we asked participants to self-report if they had made changes in their consumption of omega-3 foods, fortified products, or supplements over the course of the study (Supplementary File: Q3–Q6). When asked about consumption of omega-3 foods, there were 8, 5 and 15 individuals in the Genetic Group, and 6, 9, and 13 in the Non-Genetic group who said “*yes*”, “*no*” and “*sometimes*” to making changes to overall omega-3 consumption in their diet, respectively. Participants who said “*yes*” or “*sometimes*” were then asked a subsequent question about the factors contributing to their dietary changes.

There were 8/28 (28.6%) in the Genetic Group and 5/28 (17.9%) in the Non-Genetic group who reported that the reason for their dietary changes were related to the generic omega-3 information document provided to them at the start of the study (*p* = 0.09) (Supplementary File: Q7). However, there were 5/28 (17.9%) individuals in the Genetic group who reported that their personal genetic information was the reason they made changes to their diet. We also asked participants to identify obstacles that may have influenced their ability to change their omega-3 dietary habits (Table 4, Supplementary File: Q8). Response rates between the Genetic and Non-Genetic group were not statistically different (Table 4), although there was a trend that fewer individuals in the Genetic Group (32%) reported that “*Omega*-*3 foods are expensive*” compared to the Non-Genetic group (61%).

## 4. Discussion

This study examined the effect of providing individuals with their personal *FADS1* genetic information on the consumption of omega-3 FAs. We found that individuals in both the Genetic and Non-Genetic groups increased their intake of EPA and DHA by the end of the study, and this was reflected by significant increases in RBC %EPA. Both the Genetic and the Non-Genetic groups were meeting the minimum dietary reccomendation of 300 mg EPA and DHA/day (according to the Dietitians of Canada) by the end of the study. Providing individuals with their personal *FADS1* genetic information did not lead to significant differences in dietary intake or blood levels of omega-3 FAs compared to controls. However, the results suggest that providing individuals with genetic information can increase awareness of omega-3 FA terminology, render generic omega-3 nutritional information more useful in the context of their genetic information, and minimize barriers to the consumption of omega-3 FAs. Consequently, providing individuals with their personal *FADS1* genetic information may have an impact on longer-term omega-3 FA intake.

Our FFQ analysis revealed that Baseline intake of EPA and DHA was ~200 mg/day in our study participants, which is slightly higher than recent global reports of EPA and DHA intake (~100 mg DHA/day in developed countries) [17,18,47], although this is still lower than the minimum recommendations by Dietitians of Canada (300 mg EPA and DHA/day) [25,48]. Individuals in both the Genetic and Non-Genetic groups increased their EPA and DHA intake during the study, suggesting that providing generic omega-3 nutritional information was sufficient to motivate increased EPA and DHA consumption in young female adults. This increased dietary intake was reflected by significantly increased %EPA in RBCs. Interestingly, when examining individuals in the Genetic group more closely, minor allele carriers appeared to increase EPA and DHA consumption to a greater extent (59%) than major allele carriers (40%).

Our findings regarding the limited impact of providing personal genetic information on dietary behavior appears to conflict with previous investigations; however, important differences exist between our trial and these previous studies. For example, Hietaranta-Luoma et al. gave adults (n = 107, 20–67 years, 69% female) information about their risk for cardiovascular disease (CVD) in relation to their personal apolipoprotein E (*APOE*) genetic make-up [11]. Individuals with the highest risk for CVD showed the greatest improvements in fat quality in their diets [11]. However, our study focused on benefits to health rather than risk reduction; thus it is plausible that individuals may alter their behavior more substantially if they feel it will reduce the risk for disease instead of potentially improving health. In another study, Arkadianos et al. provided genetic information related to the Mediterranean diet to participants (*n* = 93, 46 ± 12 years, 43% women) enrolled in a weight loss program [12]. After 100 days, there were no differences between the Genetic and Non-Genetic groups; however, after ~300 days, 57% of the Genetic group maintained weight loss compared to 25% who maintained weight loss in the Non-Genetic group [12]. Therefore, it is possible that if we continued our investigation over a longer period of time, the impact of personal *FADS1* genetic information on omega-3 intake may have been more pronounced in the Genetic group compared to the Non-Genetic group.

While we did not see differences in omega-3 FA intake between the Genetic and Non-Genetic groups, it is interesting to note that participants in the Genetic group rated (using a Likert-scale) the generic omega-3 FA information document we provided at the onset of the study as new and useful (5.7/7) more often than those in the Non-Genetic group (4.9/7). This aligns with findings by Nielsen and El-Sohemy, who found that young adults who received personal genetic information related to four dietary components (caffeine, vitamin C, sodium, and sugars) reported to have a greater understanding and utility for the dietary advice provided to them [33]. Additionally, we show that participants in the Genetic group of our study reported greater awareness of omega-3 terminology after the intervention, specifically with regards to the ALA and EPA abbreviations. Similarly, when asked about barriers to omega-3 FA consumption, 61% of the Non-Genetic group rated that “*Omega*-*3 foods are expensive*” compared to 32% of the Genetic group. Interestingly, this could suggest that having personal genetic information could change attitudes about the value of healthy eating. Thus, greater awareness and a reduced perception of cost as a barrier to omega-3 intake may render these individuals more likely to choose foods with omega-3 FAs in the future.

The present study has some limitations that warrant consideration. First, the current study only included female participants who were primarily of Caucasian/European descent. Gender and ethnicity may influence dietary behavior changes upon receiving personal genetic information; therefore, a more diverse participant population is needed in future studies. Second, our population consisted solely of well-educated young adults. Future studies should examine the role of personal *FADS1* genetic information on dietary behavior changes in different subgroups of the population, such as those outside academia. Third, the FFQ used in this study was validated for EPA and DHA [30], but not ALA. Future studies should create an updated FFQ to add ALA-rich foods, fortified products, and supplements in order to better estimate the consumption of this important omega-3 FA. Since FADS1 is critical for conversion from ALA into EPA and DHA, having an estimation of ALA intake may provide more insight into differences in consumption patterns between individuals stratified according to their *FADS1* genotype. Fourth, increasing the sample size and expanding the analysis to other age groups will provide independent validation of our results. Finally, providing personalized genetic information represents a new area of investigation, therefore future research should focus on thoroughly assessing the qualitative effect of genetic information (i.e., perceptions, reactions, emotions) using focus groups.

## 5. Conclusions

The present study represents the first of its kind to explore the provision of *FADS1* genetic information and subsequent changes in omega-3 FA intake. We found little evidence that providing personal *FADS1* genetic information affected EPA and DHA intake and circulating blood levels compared to the control group. However, we did find that individuals who received their genetic information had greater awareness of omega-3 terminology, rated cost as a barrier to omega-3 consumption less often, and found their genetic information to be useful in the context of generic nutritional information pertaining to omega-3 FAs compared to the control group. Therefore, providing personal *FADS1* genetic information to young adults may provide an additional factor to help motivate behavior changes to increase the consumption of omega-3 FAs.

## Figures and Tables

**Table 1:** Demographics of the Genetic and Non-Genetic groups at Baseline.

	Genetic (*n* = 28)	Non-Genetic (*n* = 29)	χ^2^ *p*-Value
***FADS1 (rs174537) Genoptype Frequency***			
Major (GG)	13/28 (46.4%)	13/29 (44.8%)	0.79
Minor (GT + TT)	15/28 (53.6%)	16/29 (55.2%)	
***Field of study/area of work***			
Life Science	19/28 (67.9%)	21/29 (72.4%)	0.46
Social Science	7/28 (25.0%)	4/29 (13.8%)	
Other	2/28 (7.1%)	4/29 (13.8%)	
***Current Position***			
Undergraduate student	20/28 (71.4%)	19/29 (65.5%)	0.36
Graduate student	7/28 (25.0%)	8/29 (27.6%)	
Working full-time	1/28 (3.6%)	2/29 (6.9%)	
***Self*-*Reported Ethnicity***			
White/Caucasian	18/28 (64.3%)	22/29 (75.9%)	0.47
Asian	4/28 (14.3%)	3/29 (10.3%)	
European	4/28 (14.3%)	1/29 (3.5%)	
Other	2/28 (7.1%)	3/29 (10.3%)	
***Contraceptive use***			
Not taking contraceptives	8/28 (28.6%)	9/29 (31.0%)	0.89
Oral contraceptive	17/28 (60.7%)	18/29 (62.1%)	
IUD contraceptive	3/28 (10.7%)	2/29 (6.9%)	

*n* = 57 participants completed Baseline measurements and questionnaires. This data is reported as proportions (n/n total) and percentages (%). Percentages are provided in parentheses and are reported as a percentage of either the Genetic or the Non-Genetic group. Pearson’s χ^2^ analysis were conducted to determine a difference in the proportions between the Genetic and Non-Genetic groups for each parameter. *p* ≤ 0.05 was considered statistically significant. IUD, intra-uterine device.

**Table 2:** Characteristics of the Genetic and Non-Genetic groups at Baseline and Final.

	Genetic (*n* = 28)		Non-Genetic (*n* = 28)		Group	Time	Group × Time Interaction
	Baseline	Final	Baseline	Final	*p*-Value	*p*-Value	*p*-Value
***Omega*-*3 Dietary Intake (FFQ)***							
EPA and DHA (mg/day)	211.50 ± 43.16	323. 23 ± 65.27	190.16 ± 39.21	395.82 ± 70.60	0.70	**1.0 × 10^−4^**	0.27
***RBC FA levels***							
ALA (%)	0.43 ± 0.02	0.44 ± 0.02	0.41 ± 0.02	0.42 ± 0.02	0.66	0.14	0.79
EPA (%)	0.45 ± 0.02	0.51 ± 0.03	0.55 ± 0.02	0.61 ± 0.04	**3.9 × 10^−3^** *	**0.02**	0.89
DHA (%)	3.40 ± 0.11	3.42 ± 0.09	3.42 ± 0.11	3.54 ± 0.11	0.64	0.20	0.36
Omega-3 Index	3.86 ± 0.11	3.97 ± 0.11	3.97 ± 0.12	4.15 ± 0.13	0.34	**0.04**	0.66
***Clinical Data***							
BMI (kg/m^2^)	23.01 ± 0.60	22.87 ± 0.61	23.41 ± 0.50	23.55 ± 0.53	0.50	0.99	0.11
TAG (mmol/L)	0.90 ± 0.07	1.02 ± 0.08	1.02 ± 0.07	1.02 ± 0.06	0.24	0.06	0.06
Cholesterol (mmol/L)	4.40 ± 0.19	4.67 ± 0.20	4.43 ± 0.14	4.72 ± 0.14	0.87	**2.0 × 10^−4^** *	0.85
HDL (mmol/L)	1.76 ± 0.06	1.75 ± 0.07	1.75 ± 0.09	1.78 ± 0.09	0.94	0.66	0.48
Chol/HDL ratio	2.58 ± 0.13	2.70 ± 0.11	2.67 ± 0.12	2.77 ± 0.12	0.60	**0.04**	0.81
LDL (mmol/L)	2.18 ± 0.15	2.31 ± 0.14	2.22 ± 0.13	2.47 ± 0.12	0.58	**2.6 × 10^−3^** *	0.32
Non-HDL Chol	2.65 ± 0.18	2.88 ± 0.18	2.68 ± 0.13	2.94 ± 0.12	0.83	**2.0 × 10^−4^** *	0.92
***Questionnaire Data (Omega*-*3 terminology)***							
Alpha-linolenic acid	16/28 (57.1%)	23/28 (82.1%)	18/29 (62.1%)	20/28 (71.%)	0.73	**2.0 × 10^−3^** *	0.33
Eicosapentaenoic acid	12/28 (42.9%)	22/28 (78.6%)	12/29 (41.4%)	13/28 (46.4%)	0.08	**1.0 × 10^−3^** *	0.07
Docosahexaenoic acid	14/28 (50.0%)	22/28 (78.6%)	12/29 (41.4%)	14/28 (50.0%)	**0.04**	**5.0 × 10^−3^** *	0.21
ALA	12/28 (42.9%)	23/28 (82.1%)	19/29 (65.5%)	20/28 (71.4%)	0.71	**0.01** *	**0.04**
EPA	15/28 (53.6%)	23/28 (82.1%)	20/29 (69.0%)	20/28 (71.4%)	0.86	**0.01** *	**0.05**
DHA	19/28 (67.9%)	25/28 (89.3%)	19/29 (65.5%)	22/28 (78.6%)	0.46	**0.01** *	0.49

*n* = 56 participants completed Baseline and Final questionnaires (omega-3 dietary intake, questionnaire data), and blood draws for clinical data analysis. *n* = 55 participants completed Baseline and Final blood draws for RBC fatty acid (FA) data analysis. Using a ROUT outlier analysis, 4 individuals were removed from the TAG data, and 1 individual was removed from the Cholesterol, %EPA, and %ALA data. For the omega-3 dietary intake, clinical and RBC FA data, values represent mean ± SEM. The questionnaire data is represented as the proportion of participants who answered “*yes*”. Percentages are provided in parentheses. A repeated measures 2-way ANOVA was used to evaluate the effects of Group (Genetic vs. Non-Genetic) and Time (Baseline vs. Final), as well as the Group × Time interaction. *p*-values < 0.05 are shown in bold. RBC FA, clinical and questionnaire data were adjusted for multiple comparisons using a Benjamini Hochberg approach. Values significant after correction for multiple testing are indicated with a *. The question associated with omega-3 terminology data can be found in Supplementary File: Q1. ALA, alpha-linolenic acid; ANOVA, analysis of variance; BMI, body mass index; DHA, docosahexaenoic acid; EPA, eicosapentaenoic acid; FA, fatty acid; FFQ, food frequency questionnaire; HDL, high density lipoprotein cholesterol; LDL, low density lipoprotein cholesterol; ROUT, robust regression and outlier removal; SEM, standard error of mean; TAG, triglycerides.

**Table 3:** Rating of selected statements regarding the study intervention.

	Genetic (*n* = 28)	Non-Genetic (*n* = 28)	*p*-Value
	Average	Average	
I understood the nutrition information about omega-3 fats provided at the start of the study	6.21 ± 0.26	6.21 ± 0.23	1.0
The recommendations about omega-3 fats that were provided in the document at the start of the study were new to me	4.64 ± 1.75	3.71 ± 0.31	0.05
I enjoyed learning about the dietary recommendations related to omega-3 fats	6.14 ± 1.18	5.89 ± 1.07	0.41
The dietary recommendations were useful when I considered my diet throughout the study	5.68 ± 1.21	4.93 ± 1.25	0.03
When I am in the grocery store or supplement store, I can confidently determine foods that have been fortified, or have added EPA and DHA omega-3 fats	5.43 ± 1.48	5.32 ± 1.19	0.77
I would like to know more about the dietary recommendations related to omega-3 fats	5.64 ± 1.47	5.39 ± 1.47	0.53
I am interested in the relationship between diet and genetics	6.12 ± 1.45	6.61 ± 0.79	0.11

These questions were asked in the Final (week 12) study questionnaire. Participants were asked to indicate on a scale from 1–7, how much they disagreed (strongly disagreed = 1) or agreed (strongly agreed = 7) with the corresponding statements (4 was neutral). The average for each question is represented as mean ± SEM. An independent samples 2-sided *t*-test was used to determine differences between Genetic and Non-Genetic groups. A *p* < 0.05 was considered statistically significant and is indicated in bold font. The question associated with the data can be found in Supplementary File: Q2. SEM, standard error of mean.

**Table 4:** Obstacles or barriers to change diet and omega-3 FA consumption.

	Genetic (*n* = 28)	Non-Genetic (*n* = 28)	χ^2^ *p*-Value
Omega-3 foods are expensive	9/28 (32.1%)	17/28 (60.7%)	0.17
When I get busy I don’t make time to eat healthy foods	8/28 (28.6%)	4/28 (14.3%)	
I didn’t experience any barriers to change throughout this study	3/28 (10.7%)	1/28 (3.6%)	
Other obstacles/barriers ^#^	8/28 (28.6%)	6/28 (21.4%)	

These questions were asked at the end of the study in the Final questionnaire. The data is represented as the proportion of participants who answered “*yes*” to that answer option. This data is reported as proportions (n/n total) and percentages (%). Percentages are provided in parentheses and are reported as a percentage of either the Genetic or the Non-Genetic group. Pearson’s Chi-squared analysis (χ^2^) was conducted to determine a difference in the proportions between the Genetic and Non-Genetic groups. *p* ≤ 0.05 was considered statistically significant. The question associated with the data can be found in Supplementary File: Q8. ^#^ Other obstacles/barriers that could be selected by participants: It is difficult for me to get to a grocery store (Genetic *n* = 1, Non-Genetic *n* = 0); I am not involved in the grocery shopping in my home (Genetic *n* = 1, Non-Genetic *n* = 0); I eat most of my meals away from home (Genetic *n* = 0, Non-Genetic *n* = 1); I have an allergy to an omega-3 containing food (Genetic *n* = 1, Non-Genetic *n* = 1); I do not buy fortified products (Genetic *n* = 1, Non-Genetic n = 1); I do not have time to cook foods high in omega-3 fats (Genetic *n* = 2, Non-Genetic *n* = 1); I do not like fish (Genetic *n* = 1, Non-Genetic *n* = 1); I do not like taking supplements (Genetic *n* = 1, Non-Genetic *n* = 1).

## Supplementary Materials

The following are available online at http://www.mdpi.com/2072-6643/9/3/240/s1, Supplementary File: Survey Questions and Response Options.
