# Supplementary material for: Evaluating Changes in Omega-3 Fatty Acid Intake after Receiving Personal FADS1 Genetic Information: A Randomized Nutrigenetic Intervention
Source: Nutrients. 2017 Mar 6;9(3):240. doi: 10.3390/nu9030240 (PMC5372903; doi:10.3390/nu9030240)
Supplement: Supplementary file 1 [file nutrients-09-00240-s001.docx]

**Supplemental File: Survey Questions and Response Options**

**Question 1:** Before beginning this survey, had you heard of the following fats: *(Asked at Baseline and Final)*

|  | **YES** | **NO** |
| --- | --- | --- |
| ALA |  |  |
| EPA |  |  |
| DHA |  |  |
| Alpha-linolenic acid |  |  |
| Eicosapentaenoic acid |  |  |
| Docosahexaenoic acid |  |  |

**Question 2:** The following questions ask your opinion related to selected statements. Please answer these questions honestly. *(Asked at Final only)*

1 - Strongly disagree

4 – Neutral

7 - Strongly agree

1. I understood the nutrition information about omega-3 fats provided at the start of the study
2. The recommendations about omega-3 fats that were provided in the document at the start of the study were new to me
3. I enjoyed learning about the dietary recommendations related to omega-3 fats
4. The dietary recommendations were useful when I considered my diet throughout the study
5. When I am in the grocery store or supplement store, I can confidently determine foods that have been fortified, or have added EPA and DHA omega-3 fats
6. I would like to know more about the dietary recommendations related to omega-3 fats
7. I am interested in the relationship between diet and genetics

**Question 3**: Do you feel that you consciously made changes to your diet throughout this study? *(Asked at Final only)*

- Yes
- No
- Sometimes

**Question 4**: Did you choose to consume any foods high in omega-3 fats at any time since starting this study? *(Asked at Final only)*

- Yes
- No

**Question 5**: Did you choose to consume any omega-3 fortified foods or beverage products at any time since starting this study? *(Asked at Final only)*

- Yes
- No

**Question 6**: Did you choose to consume any omega-3 supplements (in capsule or liquid form) at any time since starting this study? *(Asked at Final only)*

- Yes
- No

**Question 7:** If you did make changes to your diet, what was the MOST important reason for this? *(Asked at Final only)*

- I didn’t make change to my diet
- Family medical history
- Genetic information I was given at the start of the study
- Improve my health
- Nutritional information I was given at the start of the study
- Resource file online listing products with omega-3 fats
- Took a course that discussed the health benefits of omega-3 fats

* *Note that no participants in the Non-Genetic group selected the option “Genetic information I was given at the start of the study”, as this did not apply to them.*

**Question 8**: Which factor do you think was the BIGGEST obstacle for you throughout this study? *(Asked at Final only)*

- It is difficult for me to get to a grocery store
- I am not involved in the grocery shopping in my home
- I eat the majority of my meals away from home
- Omega-3 foods are expensive
- I have an allergy to an omega-3 containing food
- I do not buy fortified products
- I do not have time to cook foods high in omega-3s
- I do not like fish
- I do not like taking supplements
- When I get busy I don't take the time to eat healthy foods
- I did not face any obstacles to increasing omega-3 intake throughout this study
